# hTERT promotes gastric intestinal metaplasia by upregulating CDX2 via NF-κB signaling pathway

**DOI:** 10.18632/oncotarget.15926

**Published:** 2017-03-06

**Authors:** Bai-Jun Chen, Shuo Zeng, Rui Xie, Chang-Jiang Hu, Su-Ming Wang, Yu-Yun Wu, Yu-Feng Xiao, Shi-Ming Yang

**Affiliations:** ^1^ Department of Gastroenterology, Xinqiao Hospital, Third Military Medical University, Chongqing, PR China; ^2^ Department of Gastroenterology, The First Affiliated Hospital, Chengdu Medical College, Chengdu, PR China

**Keywords:** hTERT, KLF4, CDX2, gastric intestinal metaplasia, NF-κB

## Abstract

**Background:**

hTERT has been reported involved in the proliferation and metastasis of gastric cancer, but the role of hTERT in gastric intestinal metaplasia, a premalignant lesion of the gastric mucosa was unknown. The aim of the present study was to investigate the role of hTERT in GIM and the effect of hTERT on CDX2 expression in gastric cells.

**Results:**

Experiments showed that expression of hTERT was significantly higher in GIM than in normal gastric mucosa. Moreover, hTERT increased the KLF4 level via NF-κB during GIM. Furthermore, KLF4 is involved in the up-regulation of CDX2 induced by hTERT, and hTERT can interact with p50, thereby increasing the level of CDX2.

**Materials and Methods:**

Immunohistochemistry was used to detect the expression of hTERT in gastric intestinal metaplasia tissue. Then, effect of hTERT on the expression of CDX2 was detected by qRT-PCR, WB and dual luciferase experiment. The role of p65 and p50 in the regulation of CDX2 were further detected by WB, CO-IP and ChIP.

**Conclusions:**

We may conclude that hTERT promotes GIM by up-regulating CDX2 via NF-κB signaling pathway.

## INTRODUCTION

Gastric cancer (GC) is the second most common malignant cancer and the fourth leading cause of cancer-related deaths worldwide [1]. In China, approximately three hundred thousand cases of gastric cancer are diagnosed annually, and GC has become a major public health threat [2, 3]. GC is a multistep and slow progressing disease whose stages include chronic gastritis, intestinal metaplasia, hyperplasia, early gastric cancer and advanced cancer. As a precancerous lesion of the stomach, gastric intestinal metaplasia (GIM) has attracted much attention in recent years [4, 5].

In GIM, normal gastric epithelium cells are replaced with intestinal cell types [6, 7]. One study suggested that the incidence of GIM advancing to gastric cancer was 1.8% in a separate cohort of patients followed up for 1 to 10 years [8]. Moreover, this study showed that the rate was higher in type III GIM than that in type I non-cancerous GIM [8]. A recent study also showed that the origin of intestinal metaplasia of gastric mucosa cells is derived from basal stem cells [9]. Under normal conditions, basal stem cells could differentiate into gastric epithelial cells to maintain their active capabilities. The caudal-related homeobox gene CDX2 (caudal type homeobox 2), plays a critical role in the progression of basal stem cell differentiation [10–13]. In the normal adult gastric epithelium, the expression of CDX2 is low, but when the gastric epithelium is stimulated by adverse external factors, the expression of CDX2 could be up-regulated, leading to the formation of GIM [14–17]. However, the molecular mechanism of CDX2 up-regulation remains unclear.

Human telomerase, which consists of human telomerase associated protein (hTP1), human telomerase RNA (hTR) and human telomerase reverse transcriptase (hTERT), hTERT is a central factor based on a rate-limiting step in telomerase activity [18]. hTERT promotes the proliferation of different types of cancer [19–22]. Previously, we observed that hTERT promotes the proliferation and invasion of gastric cancer via interactions with different genes [23–25]. In addition, the suppression of hTERT could also inhibit the malignant activity of gastric cancer [26, 27]. Previous studies have shown that the expression of hTERT in human precancerous gastric lesions was higher compared with the normal gastric epithelium, while hTR and hTP1 did not show significant changes [28]. Consistent with a previous study, there is a close association between hTERT and GIM, but the detailed mechanism needs further study.

In this paper, we investigated the role of hTERT in IM. Furthermore, we found that hTERT promotes gastric intestinal metaplasia by up-regulating CDX2 expression and its potential mechanism.

## RESULTS

### hTERT was closely related with the expression of CDX2 in GIM and gastric cancer cells

**Figure 1:**
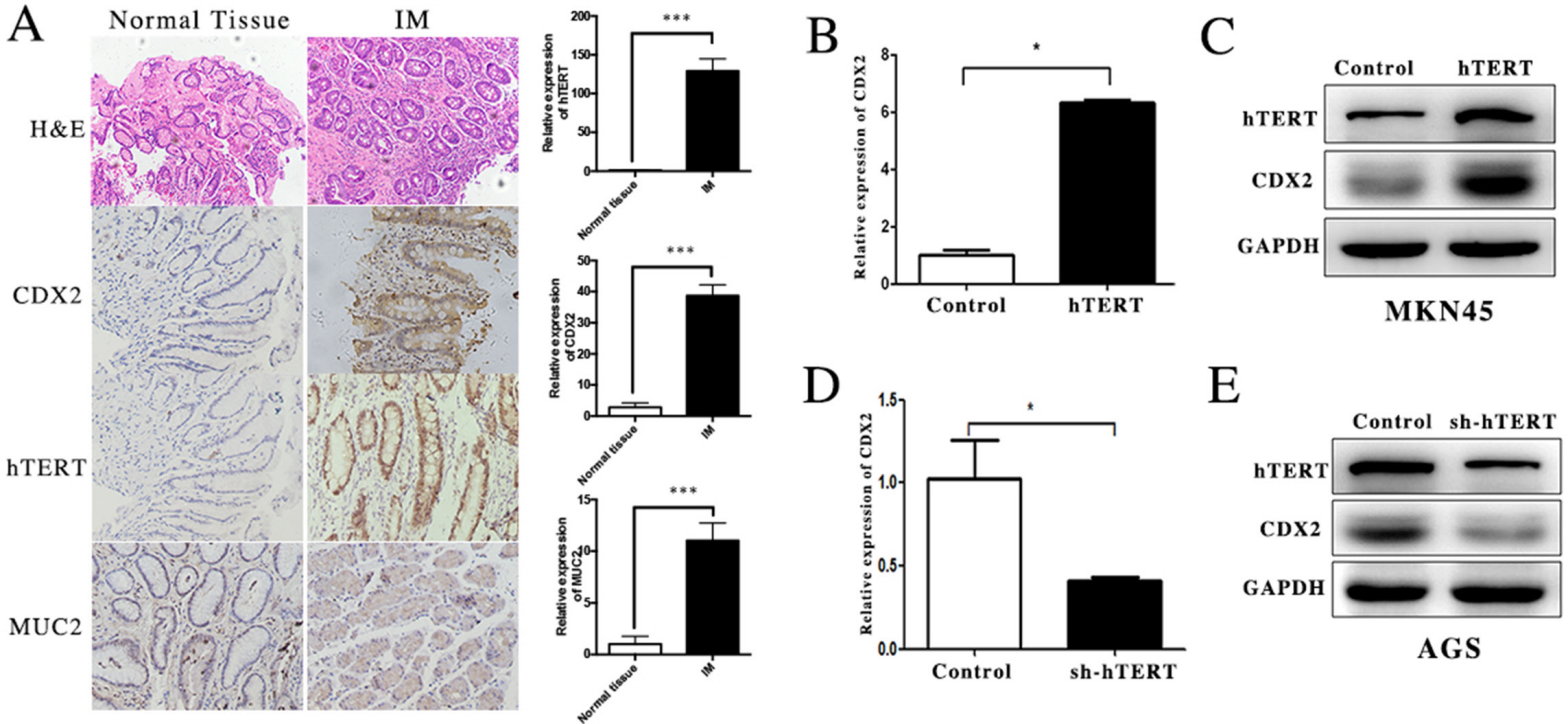
hTERT was closely related with the expression of CDX2 in GIM and gastric cancer cells (**A**) The expression of hTERT, CDX2 and MUC2 was detected in GIM tissues and normal gastric mucosa using IHC (paired *t*-test was used to analyze data, ***p < 0.001). (**B** and **C**) The expression of CDX2 in MKN45 cell was detected using qRT-PCR and WB (unpaired *t*-test was used to analyze data, *p* < 0.05). (**D** and **E**) The expression of CDX2 in AGS cell was measured using qRT-PCR and WB (unpaired *t*-test was used to analyze data,*p < 0.05).

In our study, we first detected the expression of hTERT and CDX2 in GIM tissue and corresponding normal gastric mucosa using immunohistochemistry. The expression of hTERT was increased in GIM tissues compared with that in normal gastric mucosa (Figure 1A). In addition, we measured the levels of CDX2 and its downstream gene MUC2, which was increased in GIM as a positive control (Figure 1A). We found that CDX2 and hTERT are highly expressed in GIM, which indicated that there was a strong relationship between hTERT and CDX2 in GIM tissue.

To further investigate the effect of hTERT on CDX2 expression, the hTERT over-expression plasmid was transfected into MKN45 cell line and hTERT sh-RNA plasmid was also transfected into the AGS cell line. A significant increase in CDX2 was observed at both the mRNA and protein levels after hTERT was overexpressed (Figure 1B and 1C). After suppressing the expression of hTERT in the AGS cell line, we found that the CDX2 mRNA and protein levels were decreased, as expected (Figure 1D and 1E). According to these results, hTERT could increase the expression of CDX2 at both the clinical and cellular levels.

### hTERT increase the activity of CDX2 promoter partly through NF-κB signaling pathway

**Figure 2:**
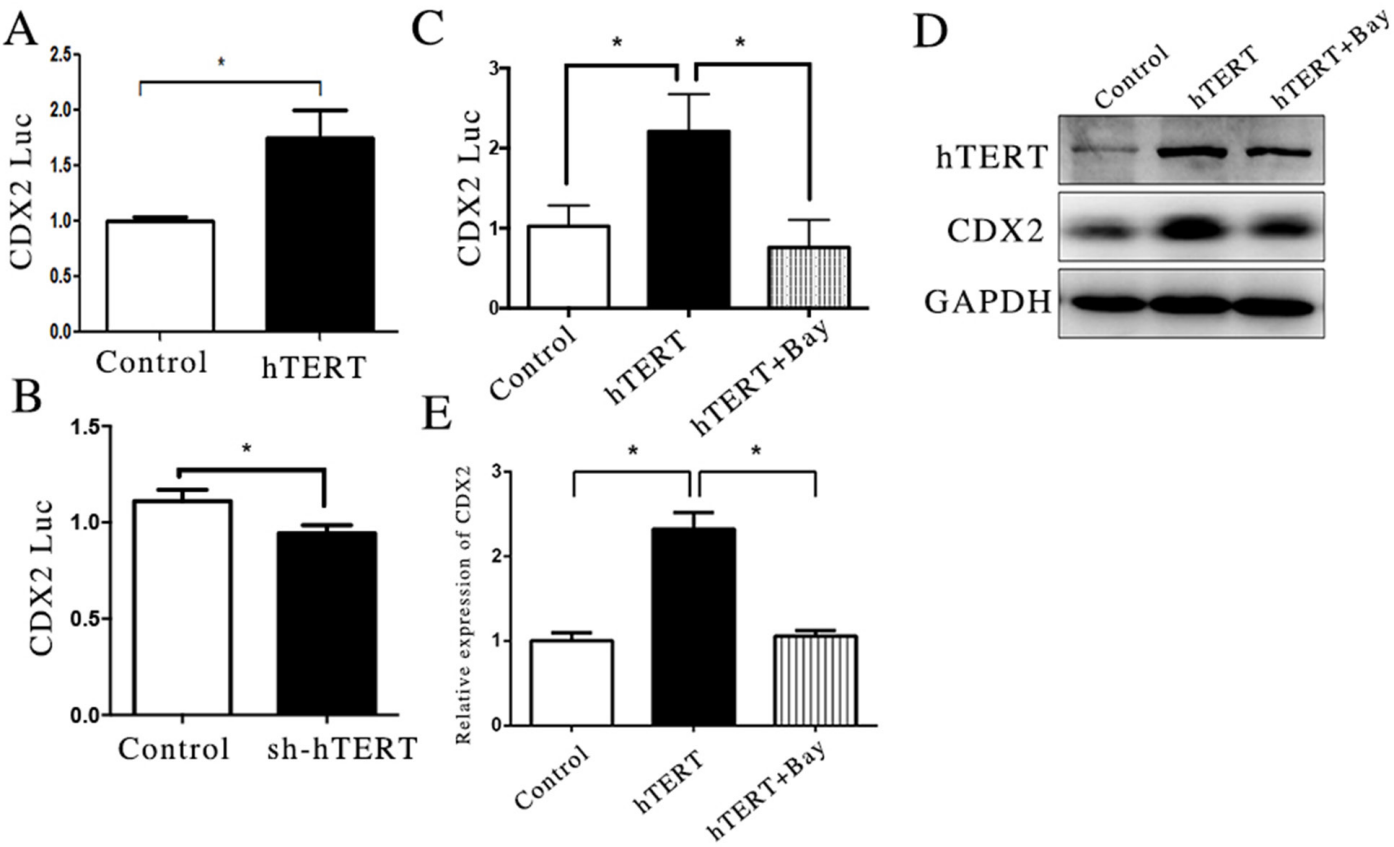
hTERT increase the activity of CDX2 promoter partly through NF-κB signaling pathway (**A** and **B**) The luciferase activity of CDX2 in MKN45 cells and AGS cells, after hTERT was over-expressed or suppressed, respectively (unpaired *t*-test was used to analyze data, *p < 0.05). (**C**) The luciferase activity of CDX2 in MKN45 cells, after the NF-κB signaling pathway was blocked (unpaired *t*-test was used to analyze data, *p < 0.05). (**D** and **E**) The mRNA and protein levels of CDX2 were measured using qRT-PCR and WB, after the NF-KB signaling pathway was blocked (unpaired *t*-test was used to analyze data, *p < 0.05).

In a previous study, we showed that the expression of CDX2 was closely related with the activity of its promoter [10]. To investigate whether the increased expression of CDX2 was induced by the activation of hTERT on the CDX2 promoter, we transfected a CDX2 reporter plasmid into MKN45 and AGS cells. The results showed that hTERT has a provoking effect on CDX2 promoter activity in transcriptional activation in the MKN45 cell line, and its depletion inhibited the activity of the CDX2 promoter (Figure 2A and 2B). Based on the present results, we concluded that hTERT could increase the expression of CDX2 by increasing CDX2 promoter. However, a recent study showed that hTERT was not a transcriptional factor, which indicated that hTERT could not directly regulate the promoter of gene. Thus, we speculated that hTERT could bind with other transcription factors to stimulate CDX2 expression.

A recent study showed the involvement of the NF-κB signaling pathway in the formation of Barrett's esophageal development [29, 30]. Moreover, recent studies have shown that hTERT could interact with the NF-κB signaling pathway [31]. According to these results, we explored the role of the NF-κB signaling pathway in GIM. Interestingly, the inhibition of NF-κB suppressed the increased CDX2 promoter activity induced by hTERT in the MKN45 cell line (Figure 2C). In addition, this phenomenon was also observed at both the CDX2 RNA and protein levels (Figure 2D and 2E). These results and those of a previous study suggest that NF-κB signaling pathway plays an important role in the up-regulation of CDX2, which is induced by hTERT. However, the detail mechanism remains unknown.

### hTERT binds to the CDX2 promoter in the gastric intestinal metaplasia cell line MKN45

**Figure 3:**
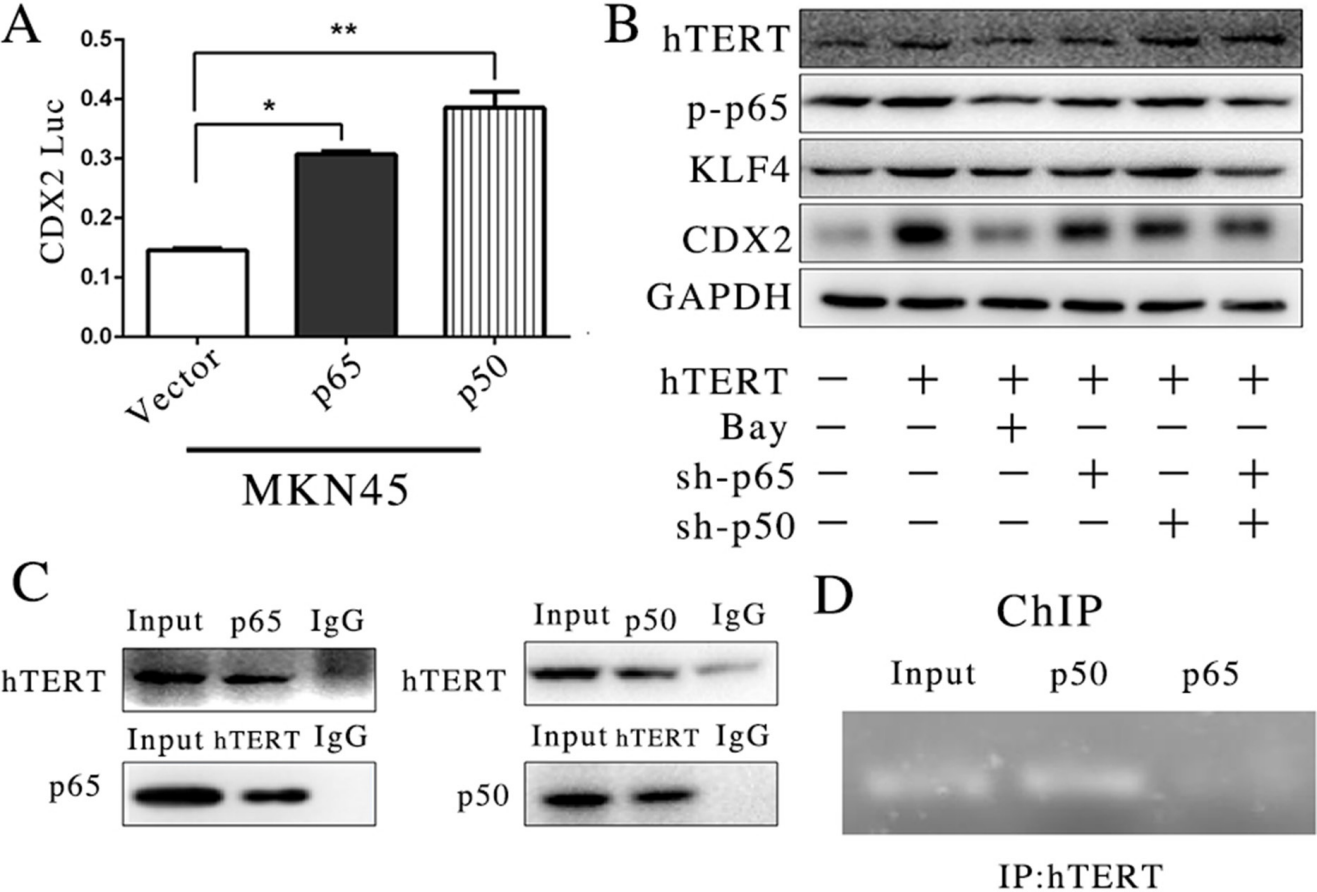
hTERT could bind with CDX2 promoter in intestinal metaplasia of gastric in the MKN45 cell line (**A**) The luciferase activity of CDX2 in MKN45 cells, after p65 and p50 was over-expressed (unpaired *t*-test was used to analyze data, *p < 0.05, **p < 0.01). (**B**) The expression of CDX2, hTERT and KLF4 was detected using WB, after p65 and p50 were suppressed or the signaling pathway was completely suppressed by Bay11-7085. (**C**) The association of hTERT with p65 or p50 was measured by Co-IP. (**D**) The ChIP assay was applied to detect the binding relationship with the CDX2 promoter.

Both p65 and p50 were important components in the NF-κB signaling pathway, and these two transcription factors have shown multiple functions in various bioactivities. According to these results, we assessed whether p65 and p50 also participated in the regulation of CDX2 promoter activity via interactions with hTERT. In certain cancer cell lines, the p65 and p50 subunits of NF-κB generate opposing effects on CDX2 expression [32, 33]. Therefore, to determine the relationship between p65/p50 and CDX2 promoter activity, we constructed p65 and p50 eukaryotic plasmids and transfected these vectors into MKN45 cells. We found that both p65 and p50 increased CDX2 promoter activity (Figure 3A). The suppression of p65 and p50 could decrease CDX2 expression at the protein level (Figure 3B). Thus, we determined whether hTERT could directly bind p65 and/or p50 to the CDX2 promoter in GIM. To confirm this hypothesis, we used a Co-IP assay to assess whether hTERT could bind with p50 and p65. Interestingly, both p65 and p50 could bind to hTERT in the MKN45 cell line (Figure 3C). Furthermore, a ChIP assay was used to detect whether p65 and p50 could bind to the CDX2 promoter. The results showed that p50 could bind to the CDX2 promoter, whereas p65 did not (Figure 3D). This result indicated that hTERT binds to p50 to confirm the complex, which could directly activate the promoter of CDX2. However, the role for p65 in the regulation of the CDX2 promoter remains unclear.

### hTERT interacts with p65 to indirectly stimulate CDX2 expression

**Figure 4:**
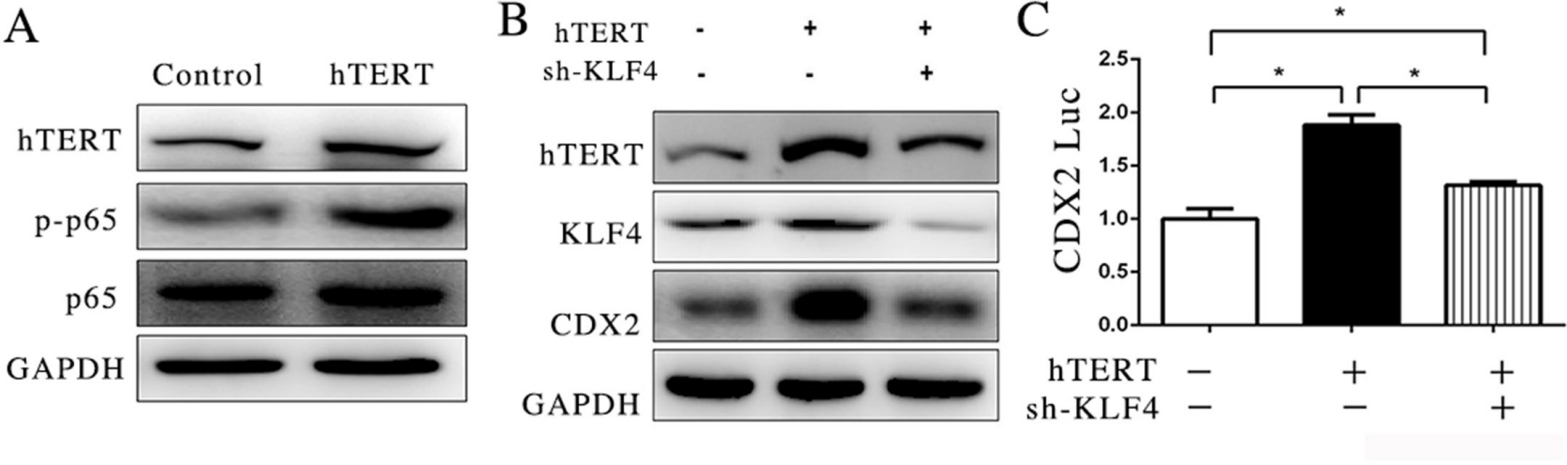
hTERT could interact with p65 to indirectly stimulate CDX2 expression (**A**) The expression of p65 and p-p65 was detected using WB after hTERT was overexpressed in MKN45 cells. (**B**) The expression of CDX2 was measured after KLF4 was suppressed. (**C**) Luciferase activity of CDX2 promoter was measured after KLF4 was suppressed (unpaired *t*-test was used to analyze data, *p < 0.05).

In the previous experiment, we found that the suppression of NF-KB signaling pathway could reduce the expression of CDX2. It has previously been reported that hTERT has a relationship with NF-κB in the nucleus and promotes the expression of NF-κB downstream gene transcription [28]. However, p65 did not bind and influence the promoter activity of CDX2 (Figure 3D). To explore the effect of hTERT on p65 protein phosphorylation in the nucleus, we used western blotting as a detection method, which showed that the over-expression of hTERT could increase phosphorylated p65 (Figure 4A), indicating that hTERT could activate p65.

KLF4 (Kruppel-like factor 4) is a transcription factor that is important in tumorigenesis. It has been reported that the KLF4 and CDX2 play an important role in Barrett's epithelium development [34]. A previous study also showed that KLF4 could be activated through p65 [27]. Thus, we wondered whether KLF4 is sufficient to promote the expression of CDX2 induced by hTERT in the GIM. To confirm this hypothesis, we transfected shRNA to reduce the expression of KLF4 in MKN45 cells. The results showed that the upregulation of CDX2 induced by hTERT could be reduced when KLF4 expression was suppressed (Figure 4B). Moreover, we found that KLF4 suppression could decrease the activity of the CDX2 promoter (Figure 4C). These results indicated that hTERT could activate phosphorylated p65 and KLF4 expression.

### hTERT activated KLF4 transcription via NF-κB pathway

**Figure 5:**
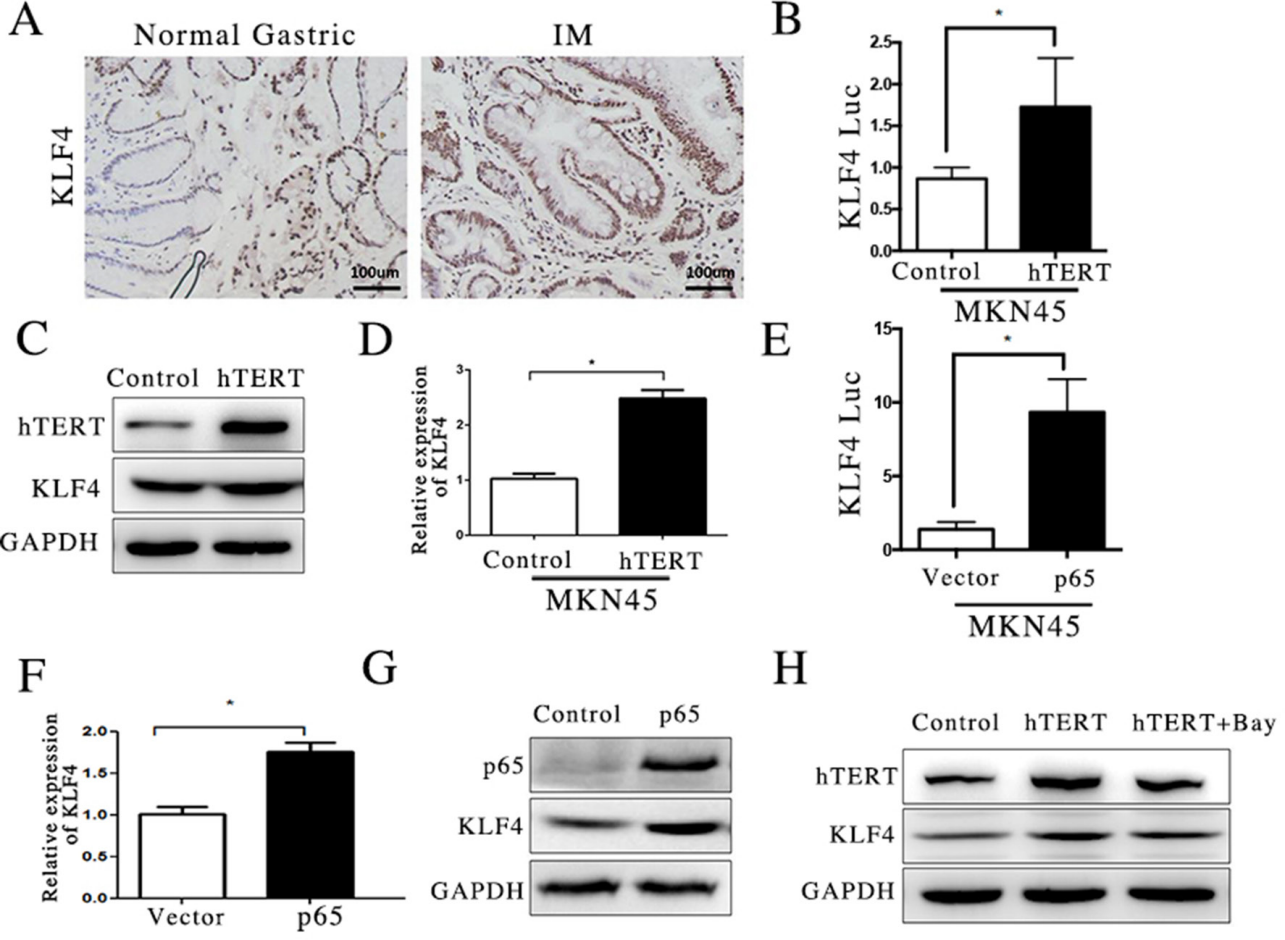
hTERT activated KLF4 transcription via NF-κB pathway (**A**) The expression of KLF4 in normal gastric mucosa and GIM was measured using IHC. (**B** and **C**) The expression of KLF4 was measured using qRT-PCR and WB after hTERT was overexpressed (unpaired *t*-test was used to analyze data, *p < 0.05). (**D**) The luciferase activity of KLF4 promoter was measured after hTERT was overexpressed (unpaired *t*-test was used to analyze data, *p < 0.05). (**E**) The luciferase activity of KLF4 was detected after p65 was overexpressed (unpaired *t*-test was used to analyze data, *p < 0.05). (**F** and **G**) The mRNA and protein levels of KLF4 were measured after p65 was overexpressed (unpaired *t*-test was used to analyze data, *p < 0.05). (**H**) The level of KLF4 was detected using WB, after NF-κB signaling pathway was suppressed by Bay11-7085.

We found that hTERT stimulated CDX2 promoter activity through KLF4 in a previous experiment. However, the role of KLF4 in GIM remains unclear. To understand the key role of KLF4 in GIM, we first used immunohistochemical staining to detect KLF4 levels in GIM tissue. KLF-positive cells with nuclear staining were clearly observed in GIM tissue (Figure 5A). Thus, we also assessed whether the overexpression of hTERT could activate the expression of KLF4. Western blotting and qRT-PCR assays were applied, showing that KLF4 mRNA and protein increased after hTERT was overexpressed in the MKN45 cell line (Figure 5B and 5C). Next, we focused on the regulatory mechanism of KLF4 induced by hTERT, we transfected a KLF4 reporter plasmid into MKN45 cells, and a Luciferase Reporter Assay was used to measure the activity of this promoter. We found that hTERT had a positive effect on KLF4 promoter activity because the activity of KLF4 promoter was highly increased (Figure 5D). A clear link between KLF4 and NF-κB pathway has been reported [27]. To examine the potential regulatory mechanism of KLF4 up-regulated by hTERT, we transfected a p65-overexpression plasmid in the MKN45 cell line and found that the overexpression of p65 increased the KLF4 expression at the mRNA, protein and promoter activity levels (Figure 5E, 5F and 5G). Moreover, we found that BAY 11-7085, an inhibitor of the NF-κB pathway, could significantly suppress the expression of KLF4 protein induced by hTERT (Figure 5H). These results suggest that hTERT activated KLF4 expression via the NF-κB pathway.

## DISCUSSION

The process of GIM is stimulated by destructive factors, such as HP infection [35–37]. In the present study, the relationship between gastric cancer and GIM is clear, particularly type III of IM, which is associated with the incidence of the aggressive form of stomach cancer [38–41]. As a premalignant lesion of gastric carcinoma, GIM has received much attention [42, 43]. However, little information is known about the process and mechanism of GIM. Thus revealing its mechanism is important in preventing the tumorigenesis of gastric cancer.

CDX2 plays a pivotal role in the differentiation of stem cells [44–47]. Under normal conditions, the expression of CDX2 was detected in IM but was absent in normal gastric cells [48]. In previous studies, researchers showed that the up-regulation of CDX2 expression during IM is important in the determination of the incomplete type of IM (type IIb, III) and early-stage GC, which had also been demonstrated in animals [49–52]. However, the regulation of CDX2 in GIM remains largely unknown.

Previous studies have suggested that hTERT serves as a critical character in the proliferation of tissue progenitor cells and human gastric cancer cells [20–23]. Interestingly, hTERT expression was higher in GIM compared with normal gastric epithelial cells. However, few studies have reported the molecular mechanism of hTERT in GIM. In this study, we detected the expression of hTERT and CDX2 at both the mRNA and protein levels in normal gastric mucosa and GIM, and hTERT expression was obviously increased in GIM (Figure 1A). In addition, the same trend was observed in the expression of CDX2 (Figure 1A).

To investigate whether hTERT could directly activate the expression of CDX2, we used qRT-PCR and western blot assays to detect the expression of CDX2, after overexpression and suppression of hTERT were procced in different cells. The results showed that hTERT could activate CDX2 expression at both the mRNA and protein levels, whereas CDX2 expression was decreased in AGS cells under knockout of hTERT (Figure 2). In addition, hTERT increased the promoter activity of CDX2 (Figure 2). These results indicated that hTERT could stimulate the level of CDX2 expression via controlling transcription activity, which promoted the process of GIM.

In addition to maintaining the elongation of telomere, Ghosh et al reported that telomerase could regulate NF-κB-dependent transcription, promoting the phosphorylation of p65 [31]. Another study reported that TERT interacts with NF-κB/p65 and activates MMP expression as a transcriptional regulator [53]. Consistent with previous studies, we showed that hTERT interacts with p65 in the nucleus and promotes the level of phosphorylated p65, which is likely associated with the suppression of degradation processes (Figure 4). These results revealed that hTERT enhanced NF-κB dependent transcription.

Study has reported that KLF4-induced activation of the CDX2-promoter is controlled by a site located between-94 and +52, by dual luciferase assay and ChIP assay in Barrett's epithelium development [29]. We also found that inhibition of KLF4 could lead to decrease of CDX2, at both protein and promoter level in our study (Figure 4), suggesting that KLF4 could influce the activity of CDX2 promoter. Subsequently, we also examined the effect of hTERT on the expression of KLF4 in our study. The results showed that both the mRNA and protein levels of KLF4 and reporter were augmented in the MKN45 cell line after transfecting hTERT (Figure 5). This result suggests that hTERT could stimulate the expression of KLF4. The promoter of KLF4 was positively regulated by p50 and p65 following exposure to bile acids in rat primary cultured keratinocytes [29]. In the present study, the NF-κB inhibitor can significantly suppress the expression of KLF4, although hTERT was over-expressed, which suggests that hTERT increased KLF4 expression via the NF-κB signaling pathway. To examine whether KLF4 increases the CDX2 expression induced by hTERT, we suppressed the expression of KLF4 and found that KLF4 could reduce the expression of CDX2, at least in part.

P65 and p50 are key factors in the NF-κB signaling pathway [54], but their function in GIM largely remained unclear [55, 56]. In the present study, we investigated the effect of p65/p50 on CDX2 expression in IM. The results showed that both p65 and p50 positively affect CDX2 promoter activity, whereas the suppression of p65 and p50 decreased the CDX2 protein levels (Figure 3). In the MKN45 cell line, only p50 could bind to the CDX2 promoter (Figure 3). These findings suggested that hTERT increases CDX2 expression through transcriptional activation via nuclear translocation and the binding of the p50 subunit of NF-κB in GIM.

**Figure 6:**
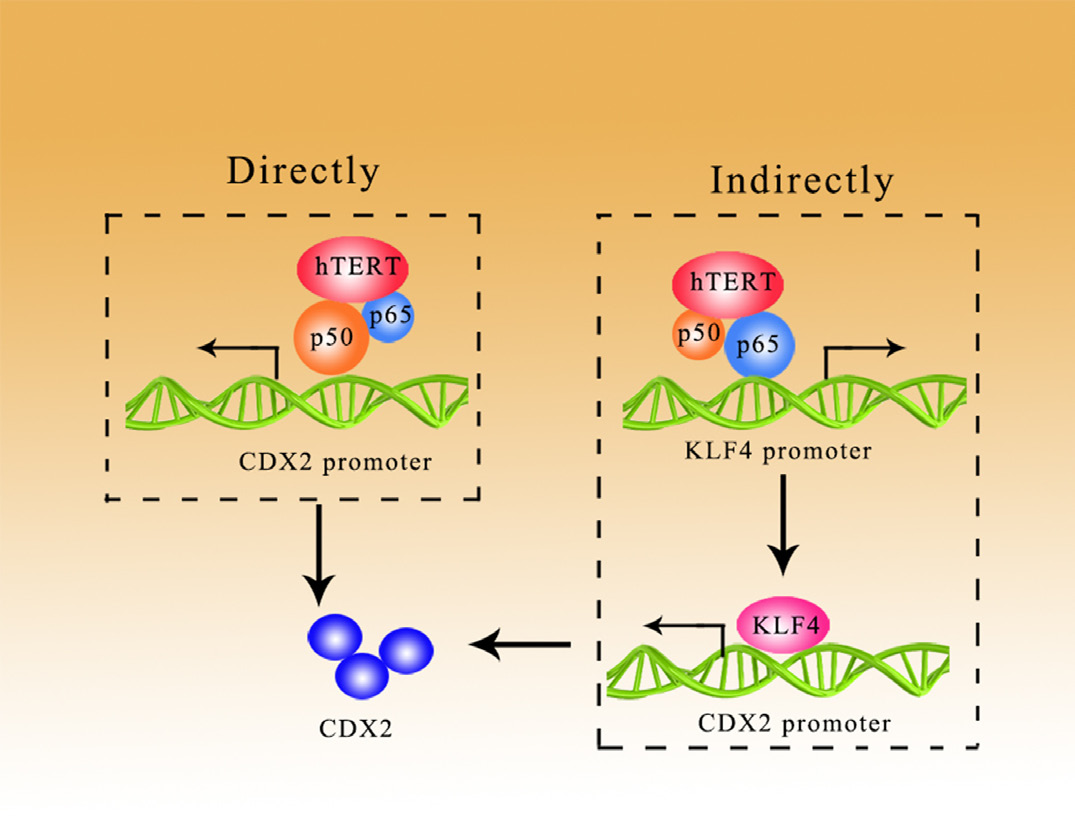
hTERT could promote the expression of CDX2 in a direct/indirect manner Direct manner: hTERT binds with p50 to increase CDX2 expression at the promoter level. Indirect manner: hTERT binds with p65 to improve the expression of KLF4, which activates the CDX2 promoter.

In conclusion, we have shown that hTERT was increased in GIM, which may promote GIM through the NF-κB signaling pathway in a direct/indirect manner. As shown in the present study, hTERT bound to p65 to improve the expression of KLF4, which could activate the CDX2 promoter; in addition, hTERT could promote the expression of CDX2 through the direct binding of p50 to the CDX2 promoter (Figure 6).

## MATERIALS AND METHODS

### Patient tissue samples

Nineteen gastric intestinal metaplasia and paired normal gastric mucosa tissue samples were obtained from the Department of Gastroenterology, the Second Affiliated Hospital of Third Military Medical University during October 2013 and July 2014. For this study, inclusion criteria were age 18–70 years, gastric intestinal metaplasia was diagnosed by one experienced endoscopic doctor, written informed consent was obtained from all patient samples. The exclusion criteria were patients with gastric cancer, coagulopathy and gastric variceal. Detailed information (such as age, sex, histological and clinical stage, and pathological data) was obtained from the hospital records. This study was approved by the Institutional Review Board of the Third Military Medical University. Two independent pathologists determined the pathological types of intestinal metaplasia according to the criteria established by classification.

### Immunohistochemistry

Gastric intestinal metaplasia and paired normal gastric epithelium tissue samples were fixed with 10% neutral formalin and embedded in paraffin, and 4-μm-thick sections were prepared. Immunostaining was performed using the avidin–biotin–peroxidase complex method (Ultrasensitive™, MaiXin, Fuzhou, China). The sections were deparaffinized in xylene, rehydrated with graded alcohol, and subsequently boiled in 0.01-M citrate buffer (pH 6.0) for 2 min in an autoclave. Hydrogen peroxide (0.3 %) was applied to block endogenous peroxide activity, and the sections were incubated with normal goat serum to reduce nonspecific binding. The tissue sections were incubated with anti-*hTERT* rabbit polyclonal antibody (1:100 dilution, Abcam, USA), anti-*CDX2* rabbit polyclonal antibody (1:100 dilution, CST, USA), and anti-*MUC2* rabbit polyclonal antibody (1:100 dilution; Abcam, USA). Rabbit immunoglobulin was used as a negative control. Staining for both antibodies was performed at room temperature for 2 h. Biotinylated goat anti-mouse serum IgG was used as a secondary antibody. After washing, the sections were incubated with streptavidin/biotin conjugated with horseradish peroxidase, and the peroxidase reaction was developed with 3, 3′-diaminobenzidine tetrahydrochloride. Counterstaining with hematoxylin was performed, and the sections were dehydrated in ethanol before mounting. The immunostaining criteria were scored on a semiquantitative scale by evaluating representative tumor areas. According to previous reports, we counted 400 cells and calculated the percentage of positively stained cells. The expression was scored as positive when >10 % of the cells in a specimen were stained. Image Pro Plus 6.0 (Media Cybernetics, USA) was used to analyze the absorbance in each sight.

### Vector construction

A total of 2881 bp of the *CDX2* promoter (Gene ID: 1045) was amplified using PCR and cloned into the Kpn I and Xho I sites in a pGL3-basic luciferase vector (Promega, Madison, WI, USA) to generate p CDX2/2881-Luc. The +1 position refers to the major transcription start site identified in the *CDX2* gene. Furthermore, 2012 bp of the *KLF4* promoter (Gene ID: 9314) was amplified using PCR, cloned into the Sac I and Hind III sites of a pGL3-basic luciferase vector to generate pKLF4/2012-Luc. Position +1 refers to the major transcription start site identified in the KLF4 gene. As an internal control for the dual luciferase assay, pRL-TATA-Renilla-Luc was used.

cDNA encoding full-length human telomerase (Gene ID: 7015) was amplified by PCR and cloned into the pIERS2-EGFP Vector (Invitrogen, Carlsbad, CA, USA). The pIERS2-EGFP Vector without *hTERT* sequences was used as a negative control, as previously reported. According to the present method, *p65* (Gene ID: 5970) and *p50* (Gene ID: 4790) expression vectors were also constructed in the M95 vector (Funeng, Guangzhou, China).

### Construction of small interfering RNA plasmid vector

Two shRNA constructs of *KLF4* were selected based on two complementary oligonucleotides

(5′-caccgaccaggcactaccgtaaacattcaagagatgtttacggta gtgcctggtctttttt-3′ and 5′-gatccaaaaaagaccaggcactaccgtaaa catctcttgaatgtttacggtagtgcctggtc-3′). Two shRNA sequences targeting *p65* were also selected: 5′-caccgcccatggaattccagtacctttcaagagaaggtactggaattccatg ggc-3′ and 5′-gatccaaaaaagcccatggaattccagtaccttctcttgaa aggtactggaattccatgggc-3′. Furthermore, two shRNA sequences for *p50* were listed as follows: 5′-caccggacagtactacctacgatggttcaagagaccatcgtaggtagtactgt ccttttttg-3′ and 5′-gatccaaaaaaggacagtactacctacgatggtctctt gaaccatcgtaggtagtactgtcc-3′. sh-RNA specific targeting *hTERT* sequence: 5′-caccgaccaggcactaccgtaaacattcaagag atgtttacggtagtgcctggtctttttt-3′ and 5′-gatccaaaaaagaccaggca ctaccgtaaacatctcttgaatgtttacggtagtgcctggtc-3′.

### Cell culture and cell transfection

Human gastric cancer cell lines MKN45 and AGS were obtained from the Type Culture Collection of the Chinese Academy of Sciences (Shanghai, China), and cultured in Dulbecco's Modified Eagle Medium (Sigma, USA) and supplemented with 10% heat-inactivated fetal bovine serum (FBS, Tianjin, China). MKN45 and AGS cells were separately cultured onto six-well plates and transfected with 2 μg of each plasmid in each well using 4 μl of Lipofectamine 200 (Invitrogen, USA), according to the manufacturer's instructions. Bay11-7085 (Sigma, USA), a pharmacologic inhibitor, was used to block IκBα phosphorylation. Cells were pretreated with Bay11-7085 at a concentration of 5 μM for 24 hours after transfection.

### RNA extraction and real-time PCR

Total RNA was extracted using the Trizol method (TaKaRa, Japan), according to a standard protocol. cDNA was synthesized using a reverse transcription kit (TaKaRa, Japan). A real-time fluorescence PCR assay based on SYBR Premix Ex Taq (TaKaRa, Japan) was then performed. Sangon Biotech synthesized the primer sequence (Sangon Biotech, China).

### Protein extraction and western blot analysis

All the proteins were extracted using RIPA buffer containing PMSF (Beyotime, China). The protein samples were loaded onto the SDS-PAGE gels and electrotransferred to PVDF membranes (Milipore, America). The membranes were blocked with 5% nonfat milk for 2 hours, and incubated overnight at 4°C with specific primary antibodies, i.e., anti-*hTERT* (1:1000, Abcam, USA), anti-*KLF4* (1:1000, Abcam, USA), anti-*p65* (1:1000, Abcam, USA), anti-*p50* (1:1000, Abcam, USA), anti- *CDX2* (1:1000, Abcam, USA), anti-*MUC2* (1:1000, Abcam, USA) and anti-*GAPDH* (1:500, Boaoshen, China) antibodies. The membranes were subsequently incubated with a secondary antibody. The bands were quantified using Quantity One software (Bio-Rad, USA), and the gray value was analyzed using GraphPad Prism software (GraphPad, USA).

### Luciferase reporter assay

Various reporter plasmids (0.1 μg) and different types of overexpression plasmids (0.1 μg) were cotransfected into MKN45 cell line. Renilla luciferase plasmids (0.1 μg) were cotransfected as an internal control for transfection efficiency. At 24 h after transfection of the luciferase vectors, a dual luciferase reporter assay (Promega, USA) was performed according to the manufacturer's instructions. Each transfection was performed in triplicate.

### Chromatin immunoprecipitation

The chromatin immunoprecipitation (Ch-IP) analysis was performed using an EpiQuik Chromatin Immunoprecipitation Kit (Epigentek Group, USA). MKN45 cells were transiently transfected with a *CDX2* promoter vector for 48 h, after which a ChIP analysis was performed. Total DNA prior to immunoprecipitation was used as the input value. Chromatin was immunoprecipitated with anti-*p50* and anti-*p65* antibodies and IgG antibody was used as a negative control. The primers were designed according to pervious study [57], and listed as follows: forward primer 5-GAGGGGTTGTGCGTAGAGTG-3 reverse primer 5-CCTTCCGTGATTAACGAGTGT-3

### Statistical analysis

The data were expressed as the means ± standard deviation (SD) and analyzed using GraphPad Prism5 software (GraphPad, America). Values were compared using Student's *t*-test or one-way ANOVA. *p* < 0.05 was considered statistically significant. Three biological replicates were applied in each experiment.
